# Interview Investigation of Insecure Attachment Styles as Mediators between Poor Childhood Care and Schizophrenia-Spectrum Phenomenology

**DOI:** 10.1371/journal.pone.0135150

**Published:** 2015-08-06

**Authors:** Tamara Sheinbaum, Antonia Bifulco, Sergi Ballespí, Mercè Mitjavila, Thomas R. Kwapil, Neus Barrantes-Vidal

**Affiliations:** 1 Departament de Psicologia Clínica i de la Salut, Universitat Autònoma de Barcelona, Barcelona, Spain; 2 Department of Psychology, Middlesex University, London, United Kingdom; 3 Department of Psychology, University of North Carolina at Greensboro, Greensboro, NC, United States of America; 4 Sant Pere Claver–Fundació Sanitària, Barcelona, Spain; 5 Centre for Biomedical Research Network on Mental Health (CIBERSAM), Instituto de Salud Carlos III, Madrid, Spain; Vanderbilt University, UNITED STATES

## Abstract

**Background:**

Insecure attachment styles have received theoretical attention and some initial empirical support as mediators between childhood adverse experiences and psychotic phenomena; however, further specificity needs investigating. The present interview study aimed to examine (i) whether two forms of poor childhood care, namely parental antipathy and role reversal, were associated with subclinical positive and negative symptoms and schizophrenia-spectrum personality disorder (PD) traits, and (ii) whether such associations were mediated by specific insecure attachment styles.

**Method:**

A total of 214 nonclinical young adults were interviewed for subclinical symptoms (Comprehensive Assessment of At-Risk Mental States), schizophrenia-spectrum PDs (Structured Clinical Interview for DSM-IV Axis II Disorders), poor childhood care (Childhood Experience of Care and Abuse Interview), and attachment style (Attachment Style Interview). Participants also completed the Beck Depression Inventory-II and all the analyses were conducted partialling out the effects of depressive symptoms.

**Results:**

Both parental antipathy and role reversal were associated with subclinical positive symptoms and with paranoid and schizotypal PD traits. Role reversal was also associated with subclinical negative symptoms. Angry-dismissive attachment mediated associations between antipathy and subclinical positive symptoms and both angry-dismissive and enmeshed attachment mediated associations of antipathy with paranoid and schizotypal PD traits. Enmeshed attachment mediated associations of role reversal with paranoid and schizotypal PD traits.

**Conclusions:**

Attachment theory can inform lifespan models of how adverse developmental environments may increase the risk for psychosis. Insecure attachment provides a promising mechanism for understanding the development of schizophrenia-spectrum phenomenology and may offer a useful target for prophylactic intervention.

## Introduction

Childhood interpersonal adversities are associated with an increased risk for psychotic disorders and subclinical psychotic phenomena [1–3]. In recent years, increasing research efforts have been devoted to identifying the underlying mechanisms that may account for such associations [4–6]. In this regard, insecure attachment styles have received theoretical attention [7] as well as some initial empirical support [8–10] as mediators between childhood adverse experiences and both positive and negative psychotic features; however, further specificity needs investigating.

Attachment theory provides an integrative approach for understanding how early relational experiences become internalized and contribute to the unfolding of adaptive or maladaptive developmental pathways [11–13]. Adult attachment researchers typically center on the construct of attachment style, which subsumes cognitive, affective, and behavioral tendencies that are considered to result from a person’s history of transactions with attachment figures [14]. The attachment style construct is useful for conceptualizing different elements associated with vulnerability for schizophrenia-spectrum psychopathology, including dysfunctional self and other representations, problems in emotion regulation, and difficulties in interpersonal functioning [7, 15]. Since the early studies in the 1990s demonstrated an association between insecure attachment styles and a diagnosis of schizophrenia (e.g., [16, 17]), evidence has accumulated showing that different forms of attachment insecurity are related to clinical and subclinical psychotic phenomena [18].

Previous work examining whether insecure attachment styles mediate the adversity–psychosis link has not specifically focused on adverse relational experiences with significant caregiving figures. This is a relevant domain of investigation given that attachment theory suggests that attachment styles are first formed in the context of the early caregiving environment [14]. Research focusing on parent-child relationships has provided evidence linking perceived lack of parental care, as well as sub-optimal parenting behaviors, with an increased likelihood of psychotic-like and schizophrenia-spectrum features (e.g., [19–21]). However, a limitation of prior studies is that most relied on self-report measures, which have potential shortcomings for researching objective aspects of early life [22].

In the present study we used the Childhood Experience of Care and Abuse (CECA) [22], an interview measure that assesses relevant context and provides objective accounts of childhood experiences, to investigate two parental behaviors within the lack of care domain: antipathy and role reversal. Antipathy reflects the extent to which the parent shows hostility, criticism, rejection or coldness towards the child. Role reversal reflects the extent to which a child assumes parental responsibilities in terms of household duties and providing emotional support to the parent [23]. A prior study using the parallel CECA questionnaire [24] reported that maternal antipathy was approximately twice as common among individuals with psychotic disorder as compared with controls [25]. To our knowledge, associations of role reversal with the extended psychosis phenotype have not been previously examined by either interview or questionnaire.

Elucidating whether there exists specificity of type of attachment style in mediating between different childhood experiences and different phenotypic expressions of psychosis should advance theory development and may ultimately inform the design of preventative and treatment strategies. Earlier studies on the role of attachment in pathways between adversity and psychotic phenomena [8–10] have relied on self-report attachment measures. Such measures are restricted in their capacity to capture the content and context of attitudinal and behavioral information. Therefore research in the field would benefit from the use of a contextually-sensitive narrative interview that provides greater specificity than questionnaire approaches for examining vulnerability to psychopathology [26, 27]. The Attachment Style Interview (ASI) [27] overcomes limitations of self-reports by using objective assessments of attachment attitudes and behaviors to identify specific attachment style profiles (encompassing secure and varieties of insecure attachment) as well as the degree of severity of the insecure styles.

Research indicates that the psychosis phenotype exists on a broad continuum that extends from schizotypic personality variation to minimal impairment to full-blown psychotic disorder and that etiological continuity appears to exist across clinical and subclinical manifestations [28, 29]. In this context, subclinical symptoms of psychosis and schizophrenia-spectrum personality disorder (PD) traits in nonclinical populations are presumed to reflect different expressions of liability to schizophrenia and help to delineate etiological processes as they avoid many of the confounds typically present in schizophrenia samples [30].

The present study sought to extend previous research by examining poor childhood care, attachment style, and schizophrenia-spectrum phenomenology using interview measures in a sample of nonclinical young adults. The goals were to investigate (i) whether childhood parental antipathy and role reversal are associated with subclinical positive and negative symptoms and schizophrenia-spectrum PD traits, and (ii) whether such associations are mediated by specific insecure attachment styles.

## Materials and Methods

### Ethics Statement

The study was approved by the Universitat Autónoma de Barcelona (Spain) Ethics Committee and conformed to the Helsinki Declaration. The participants in this interview study were over eighteen years of age and had full capacity to consent to participation in research. All participants provided written informed consent and were compensated for their participation.

### Participants

The data for the present study were collected as part of an ongoing longitudinal investigation examining risk for psychosis. The participants were drawn from a sample of 589 undergraduate students from the Universitat Autònoma de Barcelona who completed self-report questionnaires as part of mass-screening sessions. Usable screening data were obtained from 547 participants (42 were dropped due to invalid protocols). Of these, a subset of 339 was invited to participate in an interview study with the aim of assessing 200 individuals. Those invited to take part included 189 who had elevated scores (standard scores based upon sample norms of at least 1.0) on the positive or negative schizotypy factors derived from the Wisconsin Schizotypy Scales [31–34], the positive symptom subscale of the Community Assessment of Psychic Experiences [35], or the suspiciousness subscale of the Schizotypal Personality Questionnaire [36], and 150 randomly selected participants who had standard scores below 1.0 on each of these measures. This enrichment procedure was employed to ensure adequate representation of psychosis-proneness in the sample. A total of 214 participants completed the interview study. The mean age was 21.4 years (SD = 2.4) and 78% were women. Of the participants, 123 had elevated scores in one or more of the psychosis-proneness measures and 91 had standard scores below 1.0.

### Procedure

Participants were administered the measures described below along with other measures not used in the present study. The interviews were conducted by psychologists and advanced graduate students in clinical psychology who were trained in the administration of the measures and were unaware of participants’ scores on the screening questionnaires. Consensus meetings to discuss ratings were held regularly throughout the data collection period.

### Measures

#### Schizophrenia-spectrum phenomenology

Subclinical symptoms were measured with the Comprehensive Assessment of At-Risk Mental States (CAARMS) [37], which includes subscales assessing seven domains of the psychosis prodrome. Severity and frequency/duration for each subscale are rated from 0 to 6. The severity of subclinical positive and negative symptoms was calculated by summing the individual severity subscales within each symptom domain. Schizophrenia-spectrum PDs were assessed with the Structured Clinical Interview for DSM–IV Axis II Disorders (SCID–II) [38]. Items correspond to DSM–IV diagnostic criteria and are scored on a 3-point scale from “absent/false” to “threshold/true”. Dimensional scores were computed by summing individual item ratings for each PD.

#### Parental antipathy and role reversal

The CECA interview was used to assess antipathy and role reversal. These scales involve questioning participants about their experience with (and behavior from) parent figures or substitute parent figures prior to the age of 17. The severity of each experience is rated on a 4-point scale ranging from “marked” to “little/none”, based on specific rating rules and benchmarked thresholds. The ratings rely on objective aspects of experience rather than the individual’s subjective attitudes or emotional responses. Overall antipathy and role reversal ratings were obtained (i.e., peak rating taking into account behavior of both mother and father figure). The analyses used the continuous severity ratings of each childhood experience.

#### Attachment style

Attachment style was measured with the ASI, a semi-structured interview that assesses current attachment style based on detailed questioning of a person’s behavior and attitudes in close relationships. The interview consists of two parts that together determine the individual’s attachment profile: First, a rating of the ability to make and maintain relationships is made based on the overall quality of ongoing relationships with up to three supportive figures (including partner if applicable). Second, ratings are obtained on seven attitudinal attachment scales denoting avoidance (e.g., mistrust, constraints on closeness, self-reliance) and anxiety (e.g., fear of separation, fear of rejection, desire for company) in relationships. Further details of the ASI scoring procedure can be found elsewhere [23, 27]. The attachment profile encompasses the specific attachment style, including one secure, two anxious (enmeshed and fearful), and two avoidant (withdrawn and angry-dismissive) styles, as well as the degree to which the insecure styles are dysfunctional along a continuum of severity. For the present study, four attachment-style variables representing the levels of insecurity (i.e., markedly, moderately, or mildly insecure) of each of the four insecure styles were used for analyses. Due to low frequencies of marked and moderate insecure styles, these two scores were grouped together. Thus, each insecure attachment-style variable was scored 0 (not present), 1 (mildly insecure) or 2 (moderate-markedly, i.e., highly insecure).

#### Depressive symptoms

The Beck Depression Inventory–II (BDI–II) [39] was used to assess depressive symptoms. It contains 21 items that are rated on a 4-point scale ranging from 0 to 3. Higher scores indicate greater severity of depressive symptoms. Of the 214 participants, 212 had complete BDI-II data.

### Statistical Analyses

All analyses were performed using the Statistical Package for Social Sciences (SPSS), Version 19.0. Given that depressive symptoms show associations with poor childhood care, insecure attachment styles, and psychotic phenomena [23, 40–43], depressive symptoms were controlled for in all analyses. Partial correlations (partialling out the effects of depressive symptoms) were calculated to examine the associations of antipathy and role reversal with subclinical symptoms and schizophrenia-spectrum PD traits, as well as associations of these variables with the attachment styles. Two-tailed *p*-values of less than 0.05 were considered statistically significant and the effect size of the correlations was interpreted following Cohen’s [44] guidelines (correlations of 0.10 indicate small effect sizes, 0.30 indicate medium effect sizes, and 0.50 indicate large effect sizes). Hayes’ [45] method for assessing indirect pathways was used to examine the unique abilities of each insecure attachment style to account for the significant associations found between the childhood experiences and the psychosis phenotype variables. Parallel multiple mediation analyses were performed using PROCESS [45]. For each model, the four insecure attachment-style variables were entered simultaneously as mediators and depressive symptoms were entered as a covariate. The 95% and 99% bias-corrected confidence intervals were generated using bootstrapping with 10,000 resamples. Indirect effects were considered significant when the 95% bias-corrected confidence intervals did not include zero.

## Results

Table 1 provides descriptive data for the childhood experiences and schizophrenia-spectrum phenomenology variables. Regarding the prevalence of antipathy and role reversal, 34.1% of the sample experienced antipathy from parental figures (17.8% mild, 10.3% moderate, and 6.1% marked) and 36.9% experienced role reversal (18.7% mild, 14.5% moderate, and 3.7% marked). In terms of the prevalence of the attachment styles, 57.5% of the participants exhibited a secure attachment style, 35.0% a mildly insecure style, and 7.5% a highly insecure style. With regard to the type of insecure style, 5.6% exhibited an enmeshed style (0.9% highly and 4.7% mildly enmeshed), 15.9% a fearful style (3.3% highly and 12.6% mildly fearful), 6.5% an angry-dismissive style (2.3% highly and 4.2% mildly angry-dismissive), and 14.5% a withdrawn style (0.9% highly and 13.6% mildly withdrawn).

**Table 1:** Descriptive Data for Antipathy, Role Reversal, and Schizophrenia-Spectrum Phenomenology.

	Mean	SD	Range
**Childhood Experiences**			
Antipathy	1.57	0.91	1–4
Role Reversal	1.59	0.87	1–4
**Subclinical Symptoms**			
Positive Symptoms	1.21	2.69	0–24
Negative Symptoms	1.51	2.39	0–11
**PD Dimensional Scores**			
Paranoid Ratings	1.53	2.08	0–12
Schizotypal Ratings	1.00	1.93	0–13
Schizoid Ratings	0.90	1.54	0–8

Note: PD refers to Personality Disorder.

Table 2 displays the partial correlations (partialling out the effects of depressive symptoms) of antipathy and role reversal with schizophrenia-spectrum phenomenology. Both antipathy and role reversal were significantly associated with subclinical positive symptoms and with paranoid and schizotypal PD traits. Role reversal was also associated with subclinical negative symptoms. Following Cohen [44], effect sizes were of a small magnitude. For the sake of completeness, Table 3 shows the partial correlations of the insecure attachment styles with antipathy, role reversal, and the schizophrenia-spectrum phenomenology variables. The enmeshed style was associated with antipathy, role reversal, and with paranoid and schizotypal PD traits. The fearful style was associated with paranoid, schizotypal, and schizoid PD traits. The angry-dismissive style was associated with antipathy and with all the schizophrenia-spectrum phenomenology variables. Withdrawn attachment was associated with schizotypal and schizoid PD traits.

**Table 2:** Partial Correlations of Antipathy and Role Reversal with Schizophrenia-Spectrum Phenomenology.

	Positive Symptoms	Negative Symptoms	Paranoid Ratings	Schizotypal Ratings	Schizoid Ratings
**Antipathy**	0.22**	0.07	0.25***	0.23**	0.06
**Role Reversal**	0.14*	0.24***	0.17*	0.21**	0.10

***p < 0.001

**p < 0.01

*p < 0.05

**Table 3:** Partial Correlations of Levels of Insecurity of Attachment Style with Antipathy, Role Reversal, and Schizophrenia-Spectrum Phenomenology.

	Enmeshed	Fearful	Angry-Dismissive	Withdrawn
**Childhood Experiences**				
Antipathy	0.22**	0.04	0.27***	-0.06
Role Reversal	0.18**	0.01	0.10	-0.02
**Subclinical Symptoms**				
Positive Symptoms	0.12	0.08	0.16*	0.08
Negative Symptoms	0.08	0.09	0.17*	-0.00
**PD Dimensional Scores**				
Paranoid Ratings	0.20**	0.20**	**0.30** ***	0.01
Schizotypal Ratings	0.25***	0.22**	0.16*	0.16*
Schizoid Ratings	0.00	0.21**	0.25***	**0.35** ***

Note: PD refers to Personality Disorder.

***p <0.001

**p < 0.01

*p < 0.05

Medium effect sizes in bold.

Table 4 displays the results of the parallel multiple mediation analyses using antipathy as the independent variable and with depressive symptoms included as a covariate. Three models were tested (one for each of the dependent variables significantly associated with antipathy). The specific indirect effect of angry-dismissive attachment was significant in all models and the specific indirect effect of enmeshed attachment was significant in the models for paranoid and schizotypal PD traits. Table 5 presents the multiple mediator models with role reversal as the independent variable. Four models were tested (one for each of the dependent variables significantly associated with role reversal). None of the attachment styles were significant mediators of the associations of role reversal with subclinical positive and negative symptoms. The specific indirect effect of role reversal via enmeshed attachment was significant in the models for paranoid and schizotypal PD traits.

**Table 4:** Mediation Analyses Examining Indirect Effects of Antipathy on Schizophrenia-Spectrum Phenomenology via Enmeshed, Fearful, Angry-Dismissive, and Withdrawn Attachment.

			95% Bias-corrected Confidence Interval		99% Bias-corrected Confidence Interval	
	Raw Parameter Estimate	SE	Lower	Upper	Lower	Upper
**Positive Symptoms**						
Total effect	0.661**	0.203	0.262	1.061	0.135	1.188
Direct effect	0.461*	0.215	0.037	0.886	-0.099	1.021
Total indirect effect	0.200	0.128	-0.008	0.518	-0.082	0.639
Indirect effect via enmeshed	0.085	0.102	-0.027	0.420	-0.059	0.523
Indirect effect via fearful	0.015	0.039	-0.027	0.145	-0.045	0.201
Indirect effect via angry-dismissive	0.126*	0.076	0.007	0.319	-0.031	0.391
Indirect effect via withdrawn	-0.026	0.042	-0.179	0.013	-0.255	0.039
**Paranoid PD Ratings**						
Total effect	0.557**	0.152	0.258	0.855	0.162	0.951
Direct effect	0.211	0.149	-0.082	0.504	-0.176	0.598
Total indirect effect	0.346**	0.141	0.122	0.701	0.053	0.822
Indirect effect via enmeshed	0.128*	0.112	0.006	0.489	-0.002	0.622
Indirect effect via fearful	0.024	0.054	-0.061	0.160	-0.091	0.220
Indirect effect via angry-dismissive	0.210**	0.105	0.053	0.501	0.011	0.586
Indirect effect via withdrawn	-0.017	0.025	-0.089	0.013	-0.119	0.027
**Schizotypal PD Ratings**						
Total effect	0.462**	0.136	0.194	0.730	0.109	0.815
Direct effect	0.209	0.131	-0.050	0.468	-0.133	0.550
Total indirect effect	0.253**	0.124	0.064	0.567	0.007	0.713
Indirect effect via enmeshed	0.138*	0.116	0.008	0.503	-0.005	0.637
Indirect effect via fearful	0.024	0.055	-0.049	0.182	-0.074	0.240
Indirect effect via angry-dismissive	0.125*	0.066	0.025	0.306	-0.002	0.355
Indirect effect via withdrawn	-0.034	0.040	-0.127	0.033	-0.162	0.056

Note: PD refers to Personality Disorder. Results based on 10,000 bias-corrected bootstrap samples.

*95% CI does not include zero

**99% CI does not include zero

**Table 5:** Mediation Analyses Examining Indirect Effects of Role Reversal on Schizophrenia-Spectrum Phenomenology via Enmeshed, Fearful, Angry-Dismissive, and Withdrawn Attachment.

			95% Bias-Corrected Confidence Interval		99% Bias-Corrected Confidence Interval	
	Raw Parameter Estimate	SE	Lower	Upper	Lower	Upper
**Positive Symptoms**						
Total effect	0.441*	0.211	0.026	0.857	-0.106	0.989
Direct effect	0.302	0.211	-0.113	0.718	-0.246	0.851
Total indirect effect	0.139	0.107	-0.022	0.416	-0.074	0.515
Indirect effect via enmeshed	0.084	0.091	-0.019	0.376	-0.045	0.493
Indirect effect via fearful	0.005	0.036	-0.044	0.112	-0.069	0.162
Indirect effect via angry-dismissive	0.060	0.054	-0.014	0.203	-0.043	0.260
Indirect effect via withdrawn	-0.010	0.037	-0.136	0.034	-0.198	0.070
**Negative Symptoms**						
Total effect	0.615**	0.169	0.281	0.949	0.175	1.055
Direct effect	0.531**	0.172	0.193	0.870	0.085	0.978
Total indirect effect	0.084	0.076	-0.039	0.269	-0.086	0.338
Indirect effect via enmeshed	0.037	0.061	-0.042	0.222	-0.075	0.284
Indirect effect via fearful	0.004	0.027	-0.037	0.080	-0.058	0.114
Indirect effect via angry-dismissive	0.045	0.043	-0.010	0.167	-0.037	0.231
Indirect effect via withdrawn	-0.003	0.012	-0.040	0.012	-0.054	0.025
**Paranoid PD Ratings**						
Total effect	0.385*	0.158	0.074	0.696	-0.026	0.796
Direct effect	0.184	0.145	-0.102	0.470	-0.193	0.561
Total indirect effect	0.201*	0.116	0.029	0.499	-0.024	0.616
Indirect effect via enmeshed	0.113*	0.094	0.004	0.411	-0.018	0.537
Indirect effect via fearful	0.008	0.049	-0.079	0.123	-0.113	0.169
Indirect effect via angry-dismissive	0.086	0.076	-0.018	0.306	-0.064	0.381
Indirect effect via withdrawn	-0.007	0.022	-0.052	0.033	-0.068	0.061
**Schizotypal PD Ratings**						
Total effect	0.428**	0.140	0.152	0.703	0.064	0.791
Direct effect	0.262*	0.127	0.011	0.513	-0.069	0.593
Total indirect effect	0.166*	0.107	0.005	0.434	-0.036	0.538
Indirect effect via enmeshed	0.118*	0.098	0.005	0.424	-0.015	0.551
Indirect effect via fearful	0.008	0.050	-0.074	0.133	-0.105	0.188
Indirect effect via angry-dismissive	0.052	0.049	-0.011	0.191	-0.035	0.250
Indirect effect via withdrawn	-0.013	0.037	-0.079	0.072	-0.103	0.111

Note: PD refers to Personality Disorder. Results based on 10,000 bias-corrected bootstrap samples.

*95% CI does not include zero

**99% CI does not include zero.

## Discussion

The present study showed that parental antipathy and role reversal were associated with schizophrenia-spectrum phenomenology in a nonclinical sample of young adults. Although there is robust evidence linking interpersonal childhood adversities with psychotic features, the effects of antipathy and role reversal have been scarcely investigated. Our findings point to the relevance of considering their potential etiological significance alongside other forms of maltreatment. The current study also showed that particular insecure attachment styles served as mediators of associations of antipathy and role reversal with subclinical positive symptoms and/or paranoid and schizotypal PD traits. This suggests the existence of specific indirect pathways linking each childhood experience with subclinical psychotic phenomena and, more broadly, underscores the value of examining the role of attachment styles for understanding how different kinds of relational adversities might impact upon the risk and expression of schizophrenia-spectrum phenotypes. Furthermore, by partialling out the effects of depressive symptoms in all the analyses, the study provided a conservative test of the research questions and further demonstrated the incremental value of the attachment style construct over-and-above affective disturbances.

Before discussing the mediation findings it is important to highlight that the cross-sectional nature of this study limits the conclusions that can be drawn in terms of causality. The study provides useful information for the identification of potential explanatory mechanisms and we interpret the findings in accordance with the attachment literature, which has consistently identified adverse experiences with early caregiving figures as precursors to later attachment difficulties (for reviews, see [13, 14]). However, only longitudinal data can determine whether attachment processes are causally implicated in pathways between childhood experience and the development of psychotic phenomena.

Our results indicated that antipathy had an indirect effect on subclinical positive symptoms through angry-dismissive attachment and an indirect effect on paranoid and schizotypal PD traits through both angry-dismissive and enmeshed attachment. The angry-dismissive style is characterized by mistrust, self-reliance, and anger in relationships and has been associated with a coping style involving blame of others [23]. The enmeshed style is characterized by fear of separation and dependency in relationships and has been associated with a coping style involving blame of self [23]. Drawing from previous research, our findings could be interpreted to suggest that continued disapproval, rejection or hostility from parental figures might operate in at least two ways: First, it might foster an externalization of blame and projection of anger and hostility onto others (angry-dismissive pathway), which could potentially contribute to anomalies in the interpretation of others’ intentions, exacerbate attributional biases, and increase social avoidance. Second, antipathy might foster an internalization of blame as well as representations of the self as unworthy and likely to be abandoned (enmeshed pathway), which together with the anxiety and reliance on hyperactivating modes of stress regulation that characterize this style, may facilitate the emergence of paranoid and schizotypal features.

The study also showed that role reversal had an indirect effect on paranoid and schizotypal PD traits through enmeshed attachment. Previous work has conceptualized role reversal in childhood as an experience that, among other things, inhibits the development of autonomy, interferes with the differentiation of boundaries, and increases preoccupation with relationships [46–48]. Indeed, lack of autonomy, diffuse boundaries, and excessive preoccupation are elements of an enmeshed attachment. Although the exact way through which the enmeshed style links poor childhood care with paranoid and schizotypal PD traits remains to be fully clarified, we speculate that the relational ambivalence, self-regulatory deficits, and chronic hypervigilance associated with enmeshed/preoccupied forms of attachment [14] are likely to play a prominent role.

The fact that fearful and withdrawn attachment did not emerge as mediators does not preclude their role in the adversity–psychosis link; rather, it suggest that these styles might not be involved in pathways following from the childhood experiences measured in the current study. For example, previous self-report findings indicated that fearful attachment mediated associations between childhood trauma (a composite including emotional and physical forms of maltreatment) and psychosis-proneness [8]. It may be the case that this style is relevant in linking more severe forms of maltreatment with the psychosis phenotype, but this possibility should be examined in future studies. Another consideration that is pertinent to the issue of specificity is that our findings demonstrate the utility of distinguishing angry-dismissive from withdrawn attachment, a distinction that to our knowledge is only made by the ASI. Angry-dismissive and withdrawn are both avoidant styles that share in common features such as high self-reliance and high constraints on closeness, but are differentiated by the anger and mistrust of the former. This distinction has been previously found to be relevant for vulnerability profiling in relation to risk for affective disorders [49].

The strengths of the current study include the use of validated interview measures. In particular, our use of intensive interviews of childhood experience and attachment style allowed obtaining contextualized in-depth information that is not easily afforded through questionnaire approaches (and serves to minimize biases associated with subjective responding). As regards to limitations, in addition to the study’s cross-sectional nature, the use of a university student sample with a predominance of female participants may limit generalizability. Data from community samples with a more representative distribution of gender and age would enhance the generalizability of the findings. Research is also required in prodromal and clinical populations in order to determine whether these mechanisms operate across the psychosis continuum.

As for clinical implications, our results support previous suggestions that assessing childhood adverse experience and attachment style might inform service provision for individuals with psychosis [9, 15, 50]. Attachment-informed interventions have already been developed (e.g., the mentalization-based treatment for psychosis [51]), and might prove to be useful for ameliorating disturbances in those who have experienced poor childhood care from parental figures. In closing, given that attachment style is likely to be just one of the mechanisms through which adverse relational experiences might make the development of psychotic phenomena more likely, further research should consider examining whether and how specific attachment styles converge with other biological, psychological, and contextual characteristics in perpetuating risk for psychosis.

## References

[1] Varese F, Smeets F, Drukker M, Lieverse R, Lataster T, Viechtbauer W, et al Childhood adversities increase the risk of psychosis: a meta-analysis of patient-control, prospective- and cross-sectional cohort studies. Schizophr Bull. 2012;38: 661–671. 10.1093/schbul/sbs050 22461484 PMC3406538

[2] Matheson SL, Shepherd AM, Pinchbeck RM, Laurens KR, Carr VJ. Childhood adversity in schizophrenia: a systematic meta-analysis. Psychol Med. 2013;43: 225–238. 10.1017/S0033291712000785 22716913

[3] Velikonja T, Fisher HL, Mason O, Johnson S. Childhood trauma and schizotypy: a systematic literature review. Psychol Med. 2015;45: 947–963. 10.1017/S0033291714002086 25273151

[4] van Winkel R, van Nierop M, Myin-Germeys I, van Os J. Childhood trauma as a cause of psychosis: linking genes, psychology, and biology. Can J Psychiatry. 2013;58: 44–51. 23327756 10.1177/070674371305800109

[5] Bentall RP, de Sousa P, Varese F, Wickham S, Sitko K, Haarmans M, et al From adversity to psychosis: pathways and mechanisms from specific adversities to specific symptoms. Soc Psychiatry Psychiatr Epidemiol. 2014;49: 1011–1022. 10.1007/s00127-014-0914-0 24919446

[6] Sheinbaum T, Barrantes-Vidal N. Mechanisms mediating the pathway from environmental adversity to psychosis proneness In: Mason O, Claridge G, editors. Schizotypy: New dimensions. Oxford: Routledge; in press.

[7] Read J, Gumley A. Can attachment theory help explain the relationship between childhood adversity and psychosis? Attachment. 2008;2: 1–35.

[8] Sheinbaum T, Kwapil TR, Barrantes-Vidal N. Fearful attachment mediates the association of childhood trauma with schizotypy and psychotic-like experiences. Psychiatry Res. 2014;220: 691–693. 10.1016/j.psychres.2014.07.030 25095756

[9] Sitko K, Bentall RP, Shevlin M, O’Sullivan N, Sellwood W. Associations between specific psychotic symptoms and specific childhood adversities are mediated by attachment styles: an analysis of the National Comorbidity Survey. Psychiatry Res. 2014;217: 202–209. 10.1016/j.psychres.2014.03.019 24726818

[10] van Dam DS, Korver-Nieberg N, Velthorst E, Meijer CJ, de Haan L; For Genetic Risk and Outcome in Psychosis (GROUP). Childhood maltreatment, adult attachment and psychotic symptomatology: a study in patients, siblings and controls. Soc Psychiatry Psychiatr Epidemiol. 2014;49: 1759–1767. 10.1007/s00127-014-0894-0 24934617

[11] Bowlby J. Attachment and loss: Vol. 2. Separation: Anxiety and anger New York: Basic Books; 1973.

[12] Egeland B, Carlson EA. Attachment and psychopathology In: Atkinson L, Goldberg S, editors. Attachment issues in psychopathology and intervention. Mahwah, NJ: Lawrence Erlbaum Associates; 2004 pp. 27–48.

[13] Siegel DJ. The developing mind: How relationships and the brain interact to shape who we are 2nd ed. New York: Guilford Press; 2012.

[14] Mikulincer M, Shaver PR. Attachment in adulthood: Structure, dynamics, and change New York: Guilford Press; 2007.

[15] Berry K, Barrowclough C, Wearden A. A review of the role of adult attachment style in psychosis: unexplored issues and questions for further research. Clin Psychol Rev. 2007;27: 458–475. 17258365 10.1016/j.cpr.2006.09.006

[16] Dozier M, Stevenson AL., Lee SW, Velligan DI. Attachment organization and familiar overinvolvement for adults with serious psychopathological disorders. Dev Psychopathol. 1991;3: 475–489.

[17] Mickelson KD, Kessler RC, Shaver PR. Adult attachment in a nationally representative sample. J Pers Soc Psychol. 1997;73: 1092–1106. 9364763 10.1037//0022-3514.73.5.1092

[18] Korver-Nieberg N, Berry K, Meijer CJ, de Haan L. Adult attachment and psychotic phenomenology in clinical and non-clinical samples: a systematic review. Psychol Psychother. 2014;87: 127–154. 10.1111/papt.12010 23818184

[19] Janssen I, Krabbendam L, Hanssen M, Bak M, Vollebergh W, de Graaf R, et al Are apparent associations between parental representations and psychosis risk mediated by early trauma? Acta Psychiatr Scand. 2005;112: 372–375. 16223425 10.1111/j.1600-0447.2005.00553.x

[20] Meins E, Jones SR, Fernyhough C, Hurndall S, Koronis P. Attachment dimensions and schizotypy in a non-clinical sample. Pers Individ Dif. 2008;44: 1000–1011.

[21] McCabe KL, Maloney EA, Stain HJ, Loughland CM, Carr VJ. Relationship between childhood adversity and clinical and cognitive features in schizophrenia. J Psychiatr Res. 2012;46: 600–607. 10.1016/j.jpsychires.2012.01.023 22329951

[22] Bifulco A, Brown GW, Harris TO. Childhood Experience of Care and Abuse (CECA): a retrospective interview measure. J Child Psychol Psychiatry. 1994;35: 1419–1435. 7868637 10.1111/j.1469-7610.1994.tb01284.x

[23] Bifulco A, Thomas G. Understanding adult attachment in family relationships: Research, assessment, and intervention Abingdon, UK: Routledge; 2013.

[24] Bifulco A, Bernazzani O, Moran PM, Jacobs C. The childhood experience of care and abuse questionnaire (CECA.Q): validation in a community series. Br J Clin Psychol. 2005;44: 563–581. 16368034 10.1348/014466505X35344

[25] Fisher HL, Jones PB, Fearon P, Craig TK, Dazzan P, Morgan K, et al The varying impact of type, timing and frequency of exposure to childhood adversity on its association with adult psychotic disorder. Psychol Med. 2010;40: 1967–1978. 10.1017/S0033291710000231 20178679 PMC3272393

[26] Bifulco A. Attachment style measurement: a clinical and epidemiological perspective. Attach Hum Dev. 2002;4: 180–188. 12467510 10.1080/14616730210157501

[27] Bifulco A, Moran PM, Ball C, Bernazzani O. Adult attachment style. I: Its relationship to clinical depression. Soc Psychiatry Psychiatr Epidemiol. 2002;37: 50–59. 11931088 10.1007/s127-002-8215-0

[28] van Os J, Linscott RJ, Myin-Germeys I, Delespaul P, Krabbendam L. A systematic review and meta-analysis of the psychosis continuum: evidence for a psychosis proneness-persistence-impairment model of psychotic disorder. Psychol Med. 2009;39: 179–195. 10.1017/S0033291708003814 18606047

[29] Barrantes-Vidal N, Grant P, Kwapil TR. The role of schizotypy in the study of the etiology of schizophrenia-spectrum disorders. Schizophr Bull. 2015;41: 408–416. 10.1093/schbul/sbu191 PMC4373635 25810055

[30] Kwapil TR, Barrantes-Vidal N. Schizotypy: looking back and moving forward. Schizophr Bull. 2015;41: 366–373. 10.1093/schbul/sbu186 25548387 PMC4373633

[31] Chapman LJ, Chapman JP, Raulin ML. Scales for physical and social anhedonia. J Abnorm Psychol. 1976;85: 374–382. 956504 10.1037//0021-843x.85.4.374

[32] Chapman LJ, Chapman JP, Raulin ML. Body-image aberration in schizophrenia. J Abnorm Psychol. 1978;87: 399–407. 681612 10.1037//0021-843x.87.4.399

[33] Eckblad ML, Chapman LJ, Chapman JP, Mishlove M. The Revised Social Anhedonia Scale. Unpublished test. 1982 (copies available from T.R. Kwapil, Department of Psychology, University of North Carolina at Greensboro, P.O. Box 26170, Greensboro, NC, 27402–6170).

[34] Eckblad M, Chapman LJ. Magical ideation as an indicator of schizotypy. J Consult Clin Psychol. 1983;51: 215–225. 6841765 10.1037//0022-006x.51.2.215

[35] Stefanis NC, Hanssen M, Smirnis NK, Avramopoulos DA, Evdokimidis IK, Stefanis CN, et al Evidence that three dimensions of psychosis have a distribution in the general population. Psychol Med. 2002;32: 347–358. 11866327 10.1017/s0033291701005141

[36] Raine A. The SPQ: a scale for the assessment of schizotypal personality based on DSM-III-R criteria. Schizophr Bull. 1991;17: 555–564. 1805349 10.1093/schbul/17.4.555

[37] Yung AR, Yuen HP, McGorry PD, Phillips LJ, Kelly D, Dell'Olio M, et al Mapping the onset of psychosis: the Comprehensive Assessment of At-Risk Mental States. Aust N Z J Psychiatry. 2005;39: 964–971. 16343296 10.1080/j.1440-1614.2005.01714.x

[38] First MB, Gibbon M, Spitzer RL, Williams JBW, Benjamin LS. Structured Clinical Interview for DSM-IV Axis II Personality Disorders (SCID-II). Washington, DC: American Psychiatric Press; 1997.

[39] Beck AT, Steer RA, Brown GK. Manual for the Beck Depression Inventory-II. San Antonio, TX: Psychological Corporation; 1996.

[40] Harris AE, Curtin L. Parental perceptions, early maladaptive schemas, and depressive symptoms in young adults. Cognit Ther Res. 2002;26: 405–416.

[41] Roberts JE, Gotlib IH, Kassel JD. Adult attachment security and symptoms of depression: the mediating roles of dysfunctional attitudes and low self-esteem. J Pers Soc Psychol. 1996;70: 310–320. 8636884 10.1037//0022-3514.70.2.310

[42] Lewandowski KE, Barrantes-Vidal N, Nelson-Gray RO, Clancy C, Kepley HO, Kwapil TR. Anxiety and depression symptoms in psychometrically identified schizotypy. Schizophr Res. 2006;83: 225–235. 16448805 10.1016/j.schres.2005.11.024

[43] Smith B, Fowler DG, Freeman D, Bebbington P, Bashforth H, Garety P, et al Emotion and psychosis: links between depression, self-esteem, negative schematic beliefs and delusions and hallucinations. Schizophr Res. 2006;86: 181–188. 16857346 10.1016/j.schres.2006.06.018

[44] Cohen J. A power primer. Psychol Bull. 1992;112: 155–159. 19565683 10.1037//0033-2909.112.1.155

[45] Hayes AF. Introduction to mediation, moderation, and conditional process analysis: A regression-based approach New York: Guilford Press; 2013.

[46] Chase ND, editor. Burdened children: Theory, research and treatment of parentification Thousand Oaks, CA: Sage; 1999.

[47] Mayseless O, Bartholomew K, Henderson A, Trinke S. “I was more her mom than she was mine:” role reversal in a community sample. Fam Relat. 2004;53: 78–86.

[48] Macfie J, McElwain NL, Houts RM, Cox MJ. Intergenerational transmission of role reversal between parent and child: dyadic and family systems internal working models. Attach Hum Dev. 2005;7: 51–65. 15984085 10.1080/14616730500039663

[49] Bifulco A, Kwon J, Jacobs C, Moran PM, Bunn A, Beer N. Adult attachment style as mediator between childhood neglect/abuse and adult depression and anxiety. Soc Psychiatry Psychiatr Epidemiol. 2006;41: 796–805. 16871369 10.1007/s00127-006-0101-z

[50] Gumley AI, Taylor HE, Schwannauer M, MacBeth A. A systematic review of attachment and psychosis: measurement, construct validity and outcomes. Acta Psychiatr Scand. 2014;129: 257–274. 10.1111/acps.12172 23834647

[51] Brent BK, Holt DJ, Keshavan MS, Seidman LJ, Fonagy P. Mentalization-based treatment for psychosis: linking an attachment-based model to the psychotherapy for impaired mental state understanding in people with psychotic disorders. Isr J Psychiatry Relat Sci. 2014;51: 17–24. 24858631

